# Supplementary material for: From sequence to enzyme mechanism using multi-label machine learning
Source: BMC Bioinformatics. 2014 May 19;15:150. doi: 10.1186/1471-2105-15-150 (PMC4229970; doi:10.1186/1471-2105-15-150)
Supplement: Additional file 2 — Java code of ml2db. Additional file ml2db_code.tar.gz contains the Java source code to run the multi-label machine learning experiments and save the results to database. The code’s Javadoc is included. [file 1471-2105-15-150-S2.zip › additional file 2/ml2db/ml2db/doc/mulan/data/MultiLabelInstances.html]

MultiLabelInstances


JavaScript is disabled on your browser.


- Overview
- Package
- Class
- Use
- Tree
- Deprecated
- Index
- Help

- Prev Class
- Next Class

- Frames
- No Frames

- All Classes

- Summary:
- Nested |
- Field |
- Constr |
- Method

- Detail:
- Field |
- Constr |
- Method


mulan.data

## Class MultiLabelInstances

- java.lang.Object
- - mulan.data.MultiLabelInstances

- All Implemented Interfaces:
  :   java.io.Serializable

  ---

    

  ```
  public class MultiLabelInstances
  extends java.lang.Object
  implements java.io.Serializable
  ```

  Implements multi-label instances data set. Multi-label data are stored in Weka's
  `Instances`. The class is a convenient wrapper. The data are loaded form
  data file, checked for valid format. If hierarchy for labels is specified via
  XML meta-data file, the data file is cross-checked with XML for consistency.
    
  Applied rules:  
  - label names must be unique  
  - all labels in XML meta-data must be defined also in ARFF data set  
  - each label attribute must be nominal with binary values  
  - if labels has hierarchy, then if child labels indicates `true` of some
  data instance, then all its parent labels must indicate also `true` for that instance

  Author:
  :   Jozef Vilcek

  See Also:
  :   Serialized Form

- - ### Constructor Summary

    Constructors

    Constructor and Description

    `MultiLabelInstances(java.io.InputStream arffDataStream,
    java.io.InputStream xmlLabelsDefStream)`

    Creates a new instance of `MultiLabelInstances` data from the supplied `InputStream`
    data source.

    `MultiLabelInstances(java.io.InputStream arffDataStream,
    int numLabelAttributes)`

    Creates a new instance of `MultiLabelInstances` data from the supplied `InputStream`
    data source.

    `MultiLabelInstances(weka.core.Instances dataSet,
    LabelsMetaData labelsMetaData)`

    Creates a new instance of `MultiLabelInstances` data from existing `Instances`
    and `LabelsMetaData`.

    `MultiLabelInstances(weka.core.Instances data,
    java.lang.String xmlLabelsDefFilePath)`

    Creates a new instance of `MultiLabelInstances` data.

    `MultiLabelInstances(java.lang.String arffFilePath,
    int numLabelAttributes)`

    Creates a new instance of `MultiLabelInstances` data.

    `MultiLabelInstances(java.lang.String arffFilePath,
    java.lang.String xmlLabelsDefFilePath)`

    Creates a new instance of `MultiLabelInstances` data.
  - ### Method Summary

    Methods

    | Modifier and Type | Method and Description |
    | --- | --- |
    | `MultiLabelInstances` | `clone()` Returns a deep copy of the `MultiLabelInstances` instance. |
    | `double` | `getCardinality()` Gets the cardinality of the dataset |
    | `weka.core.Instances` | `getDataSet()` Gets underlying `Instances`, which contains all data. |
    | `int` | `getDepth(java.lang.String labelName)` Calculates the depth of a label, in the Hierarchy of the tree of labels. |
    | `java.util.Set<weka.core.Attribute>` | `getFeatureAttributes()` Gets the `Set` of feature `Attribute` instances of this `MultiLabelInstances` instance. |
    | `int[]` | `getFeatureIndices()` Gets the array with indices of feature attributes stored in underlying `Instances` data set. |
    | `java.util.Set<weka.core.Attribute>` | `getLabelAttributes()` Gets the `Set` of label `Attribute` instances of this `MultiLabelInstances` instance. |
    | `java.util.HashMap<java.lang.String,java.lang.Integer>` | `getLabelDepth()` Create a HashMap that contains every label, with its depth in the Hierarchical tree |
    | `int[]` | `getLabelDepthIndices()` Returns the depth of the labels |
    | `int[]` | `getLabelIndices()` |
    | `LabelsMetaData` | `getLabelsMetaData()` Gets the `LabelsMetaData` instance, which contains descriptive meta-data about label attributes stored in underlying `Instances` data set. |
    | `java.util.Map<java.lang.String,java.lang.Integer>` | `getLabelsOrder()` |
    | `int` | `getNumInstances()` Gets the number of instances |
    | `int` | `getNumLabels()` Gets the number of labels (label attributes) |
    | `boolean` | `hasMissingLabels(weka.core.Instance instance)` Method that checks whether an instance has missing labels |
    | `MultiLabelInstances` | `reintegrateModifiedDataSet(weka.core.Instances modifiedDataSet)` If `Instances` data set are retrieved from `MultiLabelInstances` and post-processed, modified by custom code, it can be again reintegrated into `MultiLabelInstances` if needed. |

    - ### Methods inherited from class java.lang.Object

      `equals, getClass, hashCode, notify, notifyAll, toString, wait, wait, wait`

- - ### Constructor Detail


    - #### MultiLabelInstances

      ```
      public MultiLabelInstances(java.lang.String arffFilePath,
                         int numLabelAttributes)
                          throws InvalidDataFormatException
      ```

      Creates a new instance of `MultiLabelInstances` data.
      The label attributes are assumed to be at the end of ARFF data file. The count
      is specified by parameter. Based on these attributes the `LabelsMetaData`
      are created.

      Parameters:
      :   `arffFilePath` - the path to ARFF file containing the data
      :   `numLabelAttributes` - the number of ARFF data set attributes which are labels.

      Throws:
      :   `ArgumentNullException` - if arrfFilePath is null
      :   `java.lang.IllegalArgumentException` - if numLabelAttribures is less than 2
      :   `InvalidDataFormatException` - if format of loaded multi-label data is invalid
      :   `DataLoadException` - if ARFF data file can not be loaded


    - #### MultiLabelInstances

      ```
      public MultiLabelInstances(java.io.InputStream arffDataStream,
                         int numLabelAttributes)
                          throws InvalidDataFormatException
      ```

      Creates a new instance of `MultiLabelInstances` data from the supplied `InputStream`
      data source. The data in the stream are assumed to be in ARFF format.
      The label attributes in ARFF data are assumed to be the last ones. Based on those attributes
      the `LabelsMetaData` are created.

      Parameters:
      :   `arffDataStream` - the `InputStream` data source to load data in ARFF format
      :   `numLabelAttributes` - the number of last ARFF data set attributes which are labels.

      Throws:
      :   `ArgumentNullException` - if `InputStream` data source is null
      :   `java.lang.IllegalArgumentException` - if number of labels attributes is less than 2
      :   `InvalidDataFormatException` - if format of loaded multi-label data is invalid
      :   `DataLoadException` - if ARFF data can not be loaded


    - #### MultiLabelInstances

      ```
      public MultiLabelInstances(weka.core.Instances data,
                         java.lang.String xmlLabelsDefFilePath)
                          throws InvalidDataFormatException
      ```

      Creates a new instance of `MultiLabelInstances` data.
      The Instances object and labels meta-data are loaded separately. The load failure is
      indicated by `DataLoadException`. When data are loaded, validations are applied
      to ensure consistency between ARFF data and specified labels meta-data.

      Parameters:
      :   `data` - the Instances object containing the data
      :   `xmlLabelsDefFilePath` - the path to XML file containing labels meta-data

      Throws:
      :   `java.lang.IllegalArgumentException` - if input parameters refers to non-existing files
      :   `InvalidDataFormatException` - if format of loaded multi-label data is invalid
      :   `DataLoadException` - if XML meta-data of ARFF data file can not be loaded


    - #### MultiLabelInstances

      ```
      public MultiLabelInstances(java.lang.String arffFilePath,
                         java.lang.String xmlLabelsDefFilePath)
                          throws InvalidDataFormatException
      ```

      Creates a new instance of `MultiLabelInstances` data.
      The ARFF data file and labels meta-data are loaded separately. The load failure is
      indicated by `DataLoadException`. When data are loaded, validations are applied
      to ensure consistency between ARFF data and specified labels meta-data.

      Parameters:
      :   `arffFilePath` - the path to ARFF file containing the data
      :   `xmlLabelsDefFilePath` - the path to XML file containing labels meta-data

      Throws:
      :   `ArgumentNullException` - if input parameters are null
      :   `java.lang.IllegalArgumentException` - if input parameters refers to non-existing files
      :   `InvalidDataFormatException` - if format of loaded multi-label data is invalid
      :   `DataLoadException` - if XML meta-data of ARFF data file can not be loaded


    - #### MultiLabelInstances

      ```
      public MultiLabelInstances(java.io.InputStream arffDataStream,
                         java.io.InputStream xmlLabelsDefStream)
                          throws InvalidDataFormatException
      ```

      Creates a new instance of `MultiLabelInstances` data from the supplied `InputStream`
      data source. The data in the stream are assumed to be in ARFF format.
      The labels meta data for ARFF data are retrieved separately from the different `InputStream`
      data source. The meta data are assumed to be in XML format and conform to valid schema.
      Data load load failure is indicated by `DataLoadException`. When data are loaded, validations
      are applied to ensure consistency between ARFF data and specified labels meta-data.

      Parameters:
      :   `arffDataStream` - the `InputStream` data source to load data in ARFF format
      :   `xmlLabelsDefStream` - the `InputStream` data source to load XML labels meta data

      Throws:
      :   `ArgumentNullException` - if input parameters are null
      :   `java.lang.IllegalArgumentException` - if input parameters refers to non-existing files
      :   `InvalidDataFormatException` - if format of loaded multi-label data is invalid
      :   `DataLoadException` - if XML meta-data of ARFF data can not be loaded


    - #### MultiLabelInstances

      ```
      public MultiLabelInstances(weka.core.Instances dataSet,
                         LabelsMetaData labelsMetaData)
                          throws InvalidDataFormatException
      ```

      Creates a new instance of `MultiLabelInstances` data from existing `Instances`
      and `LabelsMetaData`. The input parameters are not copied. Internally are stored only
      references.  
      The data set and labels meta data are validated against each other. Any violation of
      validation criteria result in `InvalidDataFormatException`.

      Parameters:
      :   `dataSet` - the data set with data instances in multi-label format
      :   `labelsMetaData` - the meta-data about label attributes of data set

      Throws:
      :   `java.lang.IllegalArgumentException` - if input parameters are null
      :   `InvalidDataFormatException` - if multi-label data format is not valid
  - ### Method Detail


    - #### getNumLabels

      ```
      public int getNumLabels()
      ```

      Gets the number of labels (label attributes)

      Returns:
      :   number of labels


    - #### getNumInstances

      ```
      public int getNumInstances()
      ```

      Gets the number of instances

      Returns:
      :   number of instances


    - #### getCardinality

      ```
      public double getCardinality()
      ```

      Gets the cardinality of the dataset

      Returns:
      :   dataset cardinality


    - #### getLabelIndices

      ```
      public int[] getLabelIndices()
      ```

      Returns:
      :   an array with the indices of the label attributes inside the
          Instances object


    - #### getLabelsOrder

      ```
      public java.util.Map<java.lang.String,java.lang.Integer> getLabelsOrder()
      ```

      Returns:
      :   a mapping of attribute names and their indices
          Instances object


    - #### getLabelAttributes

      ```
      public java.util.Set<weka.core.Attribute> getLabelAttributes()
      ```

      Gets the `Set` of label `Attribute` instances of
      this `MultiLabelInstances` instance.

      Returns:
      :   the Set of label Attribute instances


    - #### getFeatureIndices

      ```
      public int[] getFeatureIndices()
      ```

      Gets the array with indices of feature attributes stored in
      underlying `Instances` data set.

      Returns:
      :   an array with the indices of the feature attributes


    - #### getFeatureAttributes

      ```
      public java.util.Set<weka.core.Attribute> getFeatureAttributes()
      ```

      Gets the `Set` of feature `Attribute` instances of
      this `MultiLabelInstances` instance.

      Returns:
      :   the `Set` of feature `Attribute` instances


    - #### getLabelsMetaData

      ```
      public LabelsMetaData getLabelsMetaData()
      ```

      Gets the `LabelsMetaData` instance, which contains descriptive meta-data about
      label attributes stored in underlying `Instances` data set.

      Returns:
      :   descriptive meta-data about label attributes


    - #### getDataSet

      ```
      public weka.core.Instances getDataSet()
      ```

      Gets underlying `Instances`, which contains all data.

      Returns:
      :   underlying Instances object which contains all data


    - #### reintegrateModifiedDataSet

      ```
      public MultiLabelInstances reintegrateModifiedDataSet(weka.core.Instances modifiedDataSet)
                                                     throws InvalidDataFormatException
      ```

      If `Instances` data set are retrieved from `MultiLabelInstances` and
      post-processed, modified by custom code, it can be again reintegrated into
      `MultiLabelInstances` if needed. The underlying `LabelsMetaData` are
      modified to reflect changes in data set. The method creates new instance of
      `MultiLabelInstances` with modified data set and new meta-data.
        
      The supported changes are:  
      - remove of label `Attribute` to the existing `Instances`  
      - add/remove of `Instance` from the existing `Instances`  
      - add/remove of feature/predictor `Attribute` to the existing `Instances`

      Parameters:
      :   `modifiedDataSet` - the modified data set

      Returns:
      :   the modified data set

      Throws:
      :   `java.lang.IllegalArgumentException` - if specified modified data set is null
      :   `InvalidDataFormatException` - if multi-label data format with specified modifications is not valid


    - #### clone

      ```
      public MultiLabelInstances clone()
      ```

      Returns a deep copy of the `MultiLabelInstances` instance.

      **Overrides:**
      :   `clone` in class `java.lang.Object`


    - #### getLabelDepth

      ```
      public java.util.HashMap<java.lang.String,java.lang.Integer> getLabelDepth()
      ```

      Create a HashMap that contains every label, with its depth in the Hierarchical tree

      Returns:
      :   a HashMap that contains every label with its depth in the Hierarchical tree


    - #### getDepth

      ```
      public int getDepth(java.lang.String labelName)
      ```

      Calculates the depth of a label, in the Hierarchy of the tree of labels.
      Returns the counter of every level. We define the root node label that has the depth 1

      Parameters:
      :   `labelName` -

      Returns:
      :   the depth of a label


    - #### getLabelDepthIndices

      ```
      public int[] getLabelDepthIndices()
      ```

      Returns the depth of the labels

      Returns:
      :   the depth of the labels


    - #### hasMissingLabels

      ```
      public boolean hasMissingLabels(weka.core.Instance instance)
      ```

      Method that checks whether an instance has missing labels

      Parameters:
      :   `instance` - one instance of this dataset

      Returns:
      :   true if the instance has missing labels


- Overview
- Package
- Class
- Use
- Tree
- Deprecated
- Index
- Help

- Prev Class
- Next Class

- Frames
- No Frames

- All Classes

- Summary:
- Nested |
- Field |
- Constr |
- Method

- Detail:
- Field |
- Constr |
- Method
